# Single-cell RNA sequencing reveals PANoptosis signatures of acinar cells, macrophages, and neutrophils during injury and repair in acute pancreatitis

**DOI:** 10.3389/fcell.2026.1948502

**Published:** 2026-09-28

**Authors:** Weizhi Zhang, Feng Cao

**Affiliations:** 1 Capital Medical University, Beijing, China; 2 Department of General Surgery, Xuanwu Hospital, Capital Medical University, Beijing, China; 3 Clinical Center for Acute Pancreatitis, Capital Medical University, Beijing, China

**Keywords:** acute pancreatitis, macrophages, neutrophils, PANoptosis, single-cell RNA sequencing

## Abstract

**Background:**

Acute pancreatitis (AP) involves acinar cell injury, inflammation, and tissue remodeling. Although regulated cell death pathways contribute to AP progression, the cell type-specific features and dynamics of PANoptosis remain unclear. This study aimed to characterize PANoptosis-associated signatures in pancreatic cell populations at single-cell resolution.

**Methods:**

A caerulein plus lipopolysaccharide-induced mouse AP model was established. Pancreatic tissues from wild-type, AP, and early recovery groups were analyzed by single-cell RNA sequencing. Major cell types and subclusters were identified using marker genes. PANoptosis-related programs were evaluated using multiple scoring algorithms, pseudotime analysis, and functional enrichment. Histological and immunohistochemical analyses were performed for validation.

**Results:**

Single-cell profiling revealed extensive remodeling of the pancreatic cellular microenvironment during AP and recovery, characterized by acinar cell loss and increased macrophage and neutrophil infiltration. Acinar cell subclustering identified 12 transcriptionally distinct subpopulations, including injury-associated subsets with ductal-like features marked by Krt19, Epcam, Lcn2, and Krt7. Trajectory analysis suggested dynamic transitions from mature digestive enzyme-producing acinar states toward stress-associated and inflammatory states. PANoptosis-related signatures were most prominent in inflammatory acinar subsets, particularly Cluster 10, followed by Clusters 7, 4, and 5. Macrophage and neutrophil analyses further identified PANoptosis-high inflammatory subsets, especially macrophage Cluster 4 and neutrophil Cluster 4.

**Conclusion:**

This study establishes a single-cell atlas of AP injury and recovery, revealing cell-specific PANoptosis programs in acinar cells and inflammatory populations, highlighting PANoptosis as a potential link between acinar injury, immune activation, and pancreatic remodeling.

## Introduction

Acute pancreatitis (AP) is one of the most common gastrointestinal emergencies encountered in clinical practice. Its clinical course can range from mild, self-limiting inflammation to severe disease characterized by pancreatic necrosis, persistent organ failure, and even death (Boxhoorn et al., 2020). In AP, roughly 20% of patients progress to severe disease (with ≈15–35% mortality) (Fang et al., 2022). Treatment remains largely supportive (early fluid resuscitation and comprehensive supportive care), with no specific targeted therapy (Deng et al., 2026). Current evidence suggests that the early pathological events in acute pancreatitis primarily originate from disturbances in intracellular calcium homeostasis in acinar cells, mitochondrial dysfunction, excessive production of reactive oxygen species, premature activation of digestive zymogens, and stress-induced cell death (Lee and Papachristou, 2019; Zhang L. et al., 2025; Chen J. H. et al., 2025). These events subsequently amplify local and systemic inflammatory responses through the release of damage-associated molecular patterns and inflammatory mediators. Meanwhile, immune cells are not merely passive responders. Neutrophils promote disease progression by facilitating pancreatic enzyme activation, forming neutrophil extracellular traps, and exacerbating tissue injury, whereas macrophages undergo dynamic phenotypic transitions between pro-inflammatory states and reparative states involved in the clearance of necrotic debris and tissue regeneration (Chen H. et al., 2025; Ishqi et al., 2023; Xu et al., 2026). Therefore, characterizing acinar cell death and the temporal dynamics of macrophages and neutrophils along the injury–repair continuum is essential for understanding the progression of acute pancreatitis and the divergence of its clinical outcomes.

In recent years, PANoptosis has been proposed as an inflammatory form of programmed cell death that integrates key features of pyroptosis, apoptosis, and necroptosis, providing a new framework for understanding cell-fate determination under complex inflammatory conditions (Wang and Kanneganti, 2021; Zhang M. et al., 2025). Rather than treating these three cell-death modalities as separate processes, PANoptosis emphasizes that, in response to specific stimuli, a PANoptosome complex composed of molecules such as ZBP1, NLRP3, ASC, CASP8, RIPK1, and RIPK3 drives the interconnected activation of multiple cell-death pathways, thereby providing a more comprehensive explanation for inflammatory amplification, tissue destruction, and organ injury (Pandian and Kanneganti, 2022). Mechanistic studies have demonstrated that PANoptosis plays a critical role in infection, cytokine storms, and inflammatory tissue injury. In the context of pancreatic disease, both acinar cell pyroptosis and necroptosis have been shown to contribute to the pathogenesis and progression of acute pancreatitis (Wu et al., 2026; Li et al., 2025; Tian et al., 2025). Recent studies have further suggested that ZBP1–PANoptosome-mediated PANoptosis is closely associated with inflammatory injury in acute pancreatitis (Li et al., 2025). Collectively, these findings suggest that, compared with any single cell-death pathway, PANoptosis may more accurately reflect the complex cell-death network operating in acinar cells and inflammatory cells during acute pancreatitis.

Against this background, we employed a self-limiting mouse model of acute pancreatitis induced by intraperitoneal administration of caerulein in combination with lipopolysaccharide (LPS). Pancreatic tissues were collected before model induction and on days 1 and 4 after induction, representing the baseline state, injury phase, and early repair phase, respectively. It should be noted that classical caerulein-based models generally achieve relatively complete recovery at approximately 7 days, whereas regeneration-related programs may already be initiated by days 3–4. Moreover, LPS further intensifies the inflammatory response. Therefore, the day 4 samples in the present study are more appropriately defined as representing a “repair-initiation/early-repair” window rather than an endpoint of complete recovery. To characterize this injury-to-repair transition at single-cell resolution, we performed single-cell RNA sequencing of pancreatic tissues. We first constructed a comprehensive cellular atlas and subsequently performed targeted subpopulation analyses of acinar cells, macrophages, and neutrophils, with a particular focus on the dynamic changes in PANoptosis-related signaling. This study is therefore expected to delineate the trajectories of PANoptosis signaling in key cell populations during pancreatic injury and repair in acute pancreatitis and to provide a new molecular basis for therapeutic strategies aimed at mitigating inflammatory injury and promoting tissue recovery.

## Materials and methods

### Animals and reagents

All animal experiments were reviewed and approved by the Institutional Animal Care and Use Committee of Xuanwu Hospital of Capital Medical University. Male C57BL/6 mice aged 5–6 weeks were obtained from Beijing HFK Bioscience Co., Ltd., (Beijing, China) and allowed to acclimatize for 1 week before the experiments. Acute pancreatitis was induced by eight intraperitoneal injections of caerulein at a dose of 100 μg/kg, administered at 1-h intervals, followed by intraperitoneal administration of lipopolysaccharide (LPS; 20 mg/kg). 18 mice were assigned to each of the WT, AP, and Recovery groups (n = 6). Pancreatic tissues from three mice within the same group were pooled before tissue dissociation to generate one sequencing sample.

Pancreatic tissues were collected before model induction, on day 1, and on day 4, corresponding to baseline, acute injury, and early recovery, respectively. Day 1 was chosen to capture established acute histological injury, whereas day 4 was chosen to capture repair initiation before the more complete recovery generally observed around day 7–14 in self-limiting caerulein models. Caerulein and LPS were purchased from MeilunBio (Dalian, China). Hematoxylin-Eosin Stain Kit was purchased from Servicebio Technology (Wuhan, China).

### Tissue dissociation and preparation of single-cell suspensions

Single-cell RNA sequencing was performed by Singleron Biotechnologies. Briefly, after collection, pancreatic tissues were rinsed with Singleron’s pancreatic tissue preservation solution to remove surface blood, completely immersed in fresh preservation solution, and transported at 2 °C–8 °C. Under sterile conditions, the tissues were removed from the preservation solution, washed with precooled washing buffer, thoroughly minced, and incubated with pancreatic tissue dissociation solution. Following centrifugation and washing, the tissues were subjected to stepwise digestion in a 37 °C water bath. During digestion, the samples were gently mixed at regular intervals, and the supernatants containing dissociated cells were collected until the tissues were sufficiently dissociated. The resulting cell suspensions were filtered through a 70-μm cell strainer, centrifuged, and resuspended in DMEM supplemented with 10% fetal bovine serum. Red blood cell lysis and cell-clump removal were performed when necessary. After further washing and resuspension, cell concentration and viability were assessed using a Countstar® Rigel fluorescence cell analyzer to obtain high-quality pancreatic single-cell suspensions for subsequent single-cell sequencing.

### Single-cell RNA sequencing

The prepared single-cell suspensions were loaded onto microfluidic chips, and single-cell RNA-sequencing libraries were constructed according to the manufacturer’s protocol for the GEXSCOPE Single-Cell RNA Library Kit (Singleron Biotechnologies). Library preparation involved cell lysis, mRNA capture, labeling of individual cells with cell-specific barcodes and mRNA molecules with unique molecular identifiers (UMIs), reverse transcription of mRNA into cDNA, cDNA amplification, and fragmentation. The resulting libraries were sequenced on the MGI T7 platform using 150-bp paired-end reads. Raw sequencing reads were initially assessed and filtered using FastQC and fastp to remove low-quality reads, followed by the removal of adapter sequences and poly(A) tails using Cutadapt. After quality control, the clean reads were aligned to the mouse reference genome GRCm38 using STAR. Gene expression levels and UMI counts were quantified using featureCounts, and the resulting gene-by-cell expression matrix was used for subsequent bioinformatic analyses.

### Data preprocessing, quality control, and doublet removal

Single-cell RNA sequencing data were analyzed in the R environment. Briefly, the raw expression matrix of each sample was imported using the Read10X function in the Seurat package, and Seurat objects were generated based on the gene-by-cell expression matrices (Butler et al., 2018). The total number of UMIs, the number of detected genes, and the proportion of mitochondrial gene expression were then calculated for each cell. According to the quality control criteria, cells with total RNA counts ≥1,000, 200–8,000 detected genes, and mitochondrial gene proportion ≤20% were retained for downstream analysis. Doublets were identified separately for each sample using the DoubletFinder package (McGinnis et al., 2019).

### Data normalization, highly variable gene selection, and dimensionality reduction

After doublet removal, the single-cell expression matrix was normalized using Seurat. Gene expression levels in each cell were normalized using the LogNormalize method. Subsequently, the top 2,000 highly variable genes were selected. After all genes were centered and scaled using the ScaleData function, principal component analysis was performed based on the highly variable genes. The first 10 principal components were selected for downstream analysis. The Harmony algorithm was applied to integrate samples according to sample origin and to remove batch effects (Korsunsky et al., 2019). Based on the dimensionality reduction results, t-distributed stochastic neighbor embedding (t-SNE) and uniform manifold approximation and projection (UMAP) were further performed to visualize the distribution of cell populations in low-dimensional space.

Cell type annotation was manually performed based on canonical cell lineage marker genes. The DotPlot function was used to visualize the expression patterns of representative marker genes in each cell cluster, and clusters were annotated according to known cell markers of mouse pancreatic tissue. The major cell types included acinar cells, ductal cells, endocrine cells, T/NK cells, neutrophils, monocytes/macrophages, stellate cells, fibroblasts, endothelial cells, B/plasma cells, and MKI67-positive proliferating cells. The final annotation results were added to the metadata of the Seurat object, and the distribution characteristics of different cell types across experimental groups were visualized using UMAP and t-SNE plots.

### Inflammatory response gene set scoring analysis

To systematically evaluate the activity of the inflammatory response pathway across different cell populations and experimental groups, gene set scoring analysis was performed on the single-cell transcriptomic data based on the Hallmark inflammatory response gene set. Five complementary single-cell gene set scoring methods (Guan et al., 2025) were used to calculate inflammatory response scores for each cell, including AUCell, UCell, singscore, single-sample gene set enrichment analysis (ssGSEA), and the Add method. To integrate scores derived from different algorithms and reduce potential bias introduced by any single method, single-cell inflammatory response scores obtained from AUCell, UCell, singscore, ssGSEA, and the Add method were extracted separately. The resulting score matrices were then standardized. Specifically, each score was first scale-standardized, and the five standardized scores were summed to generate an integrated inflammatory response score, which was defined as inflammatory response scoring.

### Acinar cell subcluster analysis

To further characterize the heterogeneity of acinar cells under different experimental conditions, cells annotated as “Acinar cell” were extracted from the annotated single-cell dataset. A new Seurat object was then reconstructed based on the raw RNA count matrix of these cells to avoid potential effects of the original whole-cell clustering information on subcluster analysis. The acinar cells were subsequently subjected to normalization, highly variable gene selection, principal component analysis, and harmony-based batch correction. Similarly, the first 10 principal components were selected for downstream analysis. A resolution of 0.5 was used for acinar cell subclustering. UMAP and t-SNE were then applied for dimensionality reduction and visualization of acinar cell subclusters, showing both the overall distribution and the subcluster distribution patterns in the WT, AP, and Recovery groups.

To investigate the functional differences among acinar cell subclusters, functional enrichment analysis was performed based on upregulated marker genes in each subcluster. GO enrichment analysis was conducted using the clusterProfiler package (Yu, 2024) and the org.Mm.eg.db database, including the biological process, molecular function, and cellular component categories. Gene symbols were further converted to Entrez IDs for KEGG and HALLMARK pathway enrichment analyses.

To evaluate potential state transitions among acinar cell subclusters, pseudotime analysis was performed using Monocle2. The pseudotime trajectories were visualized according to acinar cell subclusters, State, Pseudotime, and experimental group. To further validate the potential differentiation relationships among acinar cell subclusters, trajectory inference was also performed using Slingshot. In addition, CytoTRACE was used to assess the differentiation potential of acinar cells, and CytoTRACE scores were mapped onto the dimensionality reduction results for visualization.

To evaluate the activity of cell death-related pathways in acinar cells, single-cell scoring was performed based on apoptosis, necroptosis, pyroptosis, and PANoptosis gene sets. Pyroptosis, apoptosis, necroptosis, and PANoptosis gene sets were obtained from the previous study (Yuan et al., 2023). Using an approach similar to that applied for inflammatory response scoring, five methods, including AUCell, UCell, singscore, ssGSEA, and AddModuleScore, were used to calculate single-cell activity scores for each gene set. The scores obtained from the different algorithms were then standardized and normalized, and the five standardized scores were summed to generate an integrated score.

### Macrophage and neutrophil subcluster analysis

The subcluster analysis strategy for macrophages and neutrophils was similar to that used for acinar cells. Briefly, macrophages and neutrophils were separately extracted from the complete single-cell dataset to construct new Seurat objects. Appropriate numbers of subclusters were then identified, followed by enrichment analyses to characterize the functional features of each subcluster, including BP, KEGG, and Hallmark enrichment analyses. Multiple algorithms, including AUCell, UCell, singscore, ssGSEA, and AddModuleScore, were used to evaluate PANoptosis-related signatures. The scoring is defined as the accumulated normalized score of each algorithm, where each algorithm is first normalized and then the resulting scores are summed.

### Hematoxylin and eosin (HE) staining

Paraffin-embedded tissue sections were subjected to hematoxylin and eosin (HE) staining using a commercial HE staining kit (Cat. G1120, Solarbio, China) following the manufacturer’s protocol. After staining, the sections were scanned and digitally archived using a slide scanner.

### Immunohistochemistry (IHC)

Paraffin-embedded tissue sections were baked, dewaxed, and rehydrated through a graded ethanol series. Antigen retrieval was performed using EDTA retrieval buffer, followed by PBS washing. Endogenous peroxidase activity was blocked with 3% hydrogen peroxide, and nonspecific binding was blocked with blocking buffer. The sections were then incubated overnight at 4 °C with primary antibodies against Ly6 g (Servicebio, GB12229-50, 1:1,000) and F4/80 (Servicebio, GB113373-50, 1:1,000). After washing with PBS, the sections were incubated with corresponding HRP-conjugated secondary antibodies. Immunoreactivity was visualized using DAB substrate, followed by hematoxylin counterstaining. Finally, the sections were differentiated, blued, dehydrated, cleared, mounted with neutral resin, and digitally scanned.

## Results

### Single-cell transcriptomic profiling reveals dynamic cellular remodeling during acute pancreatitis and recovery

To characterize the cellular landscape of pancreatic tissues during acute pancreatitis and recovery, we performed single-cell RNA sequencing on pancreatic samples from WT, AP, and Recovery groups. Each group contained six mice, and every three mice were pooled to generate one sequencing sample, resulting in two pooled samples per group. Histological examination showed that pancreatic tissues from the WT group displayed intact lobular architecture and well-organized acinar structures, whereas AP tissues exhibited pronounced tissue injury, acinar disruption, interstitial edema, and inflammatory cell infiltration. In the Recovery group, pancreatic tissue architecture was partially restored, although residual structural remodeling and inflammatory changes were still observed (Figure 1A).

**FIGURE 1:**
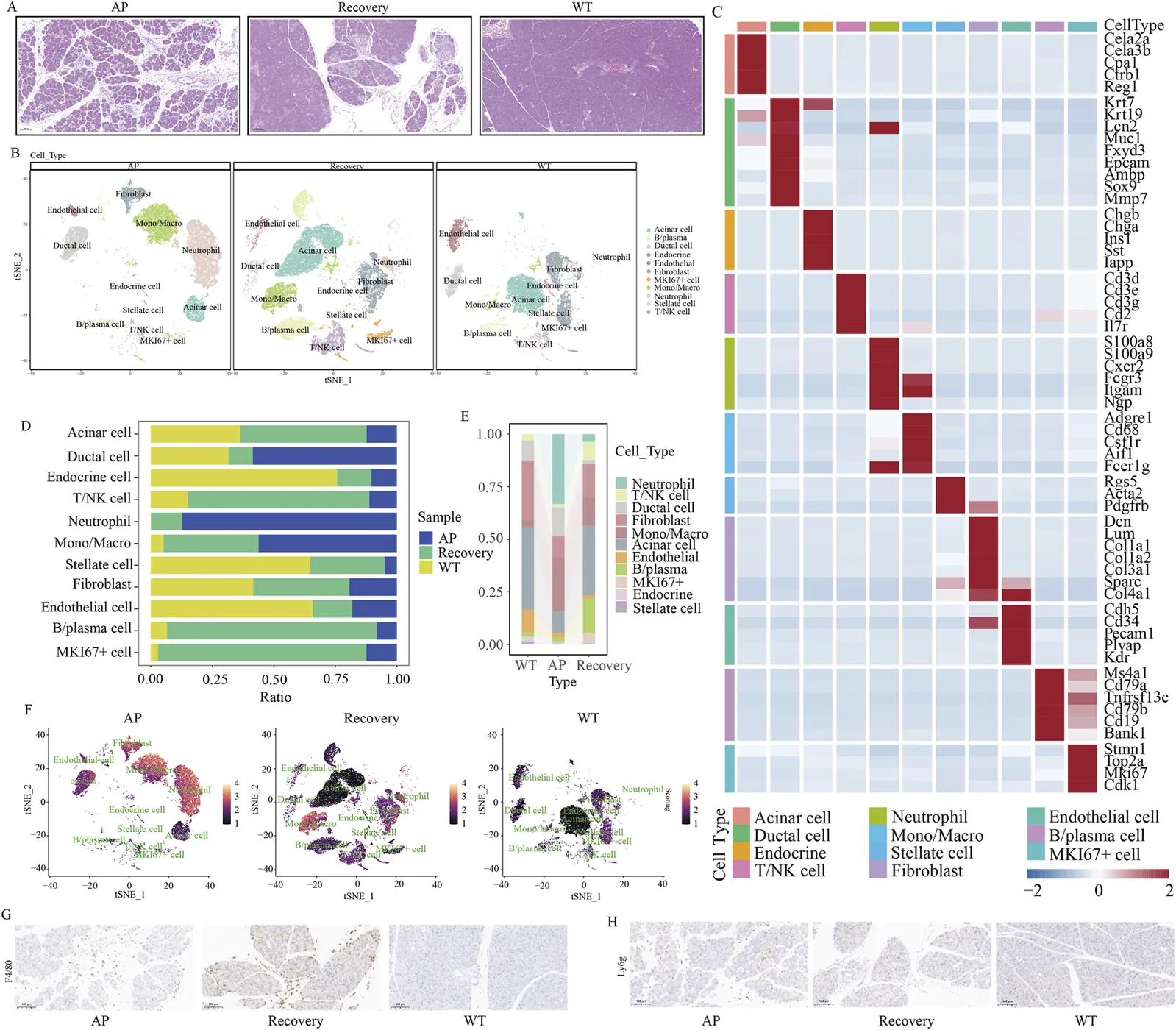
Single-cell transcriptomic landscape of pancreatic tissues during acute pancreatitis and recovery. **(A)** Representative HE staining images of pancreatic tissues from the acute pancreatitis (AP), recovery, and wild-type (WT) groups. **(B)** t-SNE visualization of all high-quality single cells after quality control and doublet removal, colored by annotated cell types and split by experimental group. **(C)** Heatmap showing the expression of representative marker genes used for cell type annotation. **(D)** Relative proportions of WT, AP, and Recovery cells within each annotated cell type. **(E)** Stacked bar plot showing the overall cellular composition of pancreatic tissues in the WT, AP, and Recovery groups. **(F)** t-SNE plots showing inflammatory response scores across different experimental groups. **(G)** Representative immunohistochemical staining images of F4/80-positive macrophages in pancreatic tissues from the AP, Recovery, and WT groups. **(H)** Representative immunohistochemical staining images of Ly6g-positive neutrophils in pancreatic tissues from the AP, Recovery, and WT groups.

After initial sequencing, a total of 77,445 cells were obtained from all samples. Following quality control filtering, 64,090 high-quality cells were retained. DoubletFinder was then applied to remove potential doublets, resulting in 58,804 single cells for downstream analysis, including 19,296 cells from the AP group, 24,819 cells from the Recovery group, and 14,689 cells from the WT group. Unsupervised clustering and t-SNE visualization identified distinct cellular populations across pancreatic tissues (Figure 1B). Based on canonical marker gene expression, these clusters were annotated into 11 major cell types, including acinar cells, ductal cells, endocrine cells, T/NK cells, neutrophils, monocytes/macrophages, stellate cells, fibroblasts, endothelial cells, B/plasma cells, and MKI67^+^ proliferating cells. The reliability of cell type annotation was further confirmed by the expression patterns of representative marker genes (Figure 1C). For example, Cela2a, Cela3b, Cpa1, Ctrb1, and Reg1 marked acinar cells; Krt7, Krt19, Muc1, Epcam, and Sox9 characterized ductal cells; S100a8, S100a9, Cxcr2, and Itgam were enriched in neutrophils; and Adgre1, Cd68, Csf1r, and Aif1 defined monocytes/macrophages. We next compared the cellular composition across different groups. The relative distribution of each cell type varied substantially across disease stages (Figures 1D,E).

Compared with WT tissues, AP tissues showed a marked increase in inflammatory cell populations, particularly neutrophils and monocytes/macrophages, accompanied by a relative reduction in acinar cells. In the Recovery group, the cellular composition partially shifted toward the WT pattern, although immune and stromal cell populations remained altered, suggesting incomplete resolution of inflammation and ongoing tissue remodeling during recovery. To further evaluate inflammatory activation at the single-cell level, inflammatory response scores were mapped onto the t-SNE space. The AP group exhibited stronger inflammatory signaling than the WT group, with high scores mainly localized to immune-cell-enriched regions. Inflammatory activity was reduced in the Recovery group compared with AP, but remained detectable in several cell populations, indicating persistent inflammatory remodeling during the recovery phase (Figure 1F).

Finally, immunohistochemical staining was performed to validate the infiltration of macrophages and neutrophils in pancreatic tissues. F4/80 staining showed increased macrophage accumulation in AP tissues and persistent macrophage presence in the Recovery group compared with WT tissues (Figure 1G). Ly6G staining showed pronounced neutrophil infiltration in AP tissues, with lower but still detectable staining during early recovery (Figure 1H). Together, these results demonstrate that acute pancreatitis induces extensive remodeling of the pancreatic cellular microenvironment, characterized by acinar injury, immune cell infiltration, and partial but incomplete restoration during recovery.

### Acinar cell subclustering reveals marked heterogeneity and ductal-like transition during acute pancreatitis

To further investigate the heterogeneity of acinar cells during acute pancreatitis and recovery, acinar cells were extracted from the integrated single-cell dataset and subjected to secondary clustering analysis. Unsupervised clustering identified 12 acinar cell subclusters, which displayed distinct distributions among the AP, Recovery, and WT groups (Figure 2A). WT samples were mainly enriched in clusters with typical acinar features, whereas AP and Recovery samples contained several additional acinar subclusters, suggesting disease-associated remodeling of acinar cell states. The group composition of each acinar subcluster further confirmed that different subclusters showed distinct sample preferences across WT, AP, and Recovery conditions (Figure 2B). Differential expression analysis revealed specific marker genes for each acinar subcluster, indicating substantial transcriptional heterogeneity within acinar cells (Figure 2C).

**FIGURE 2:**
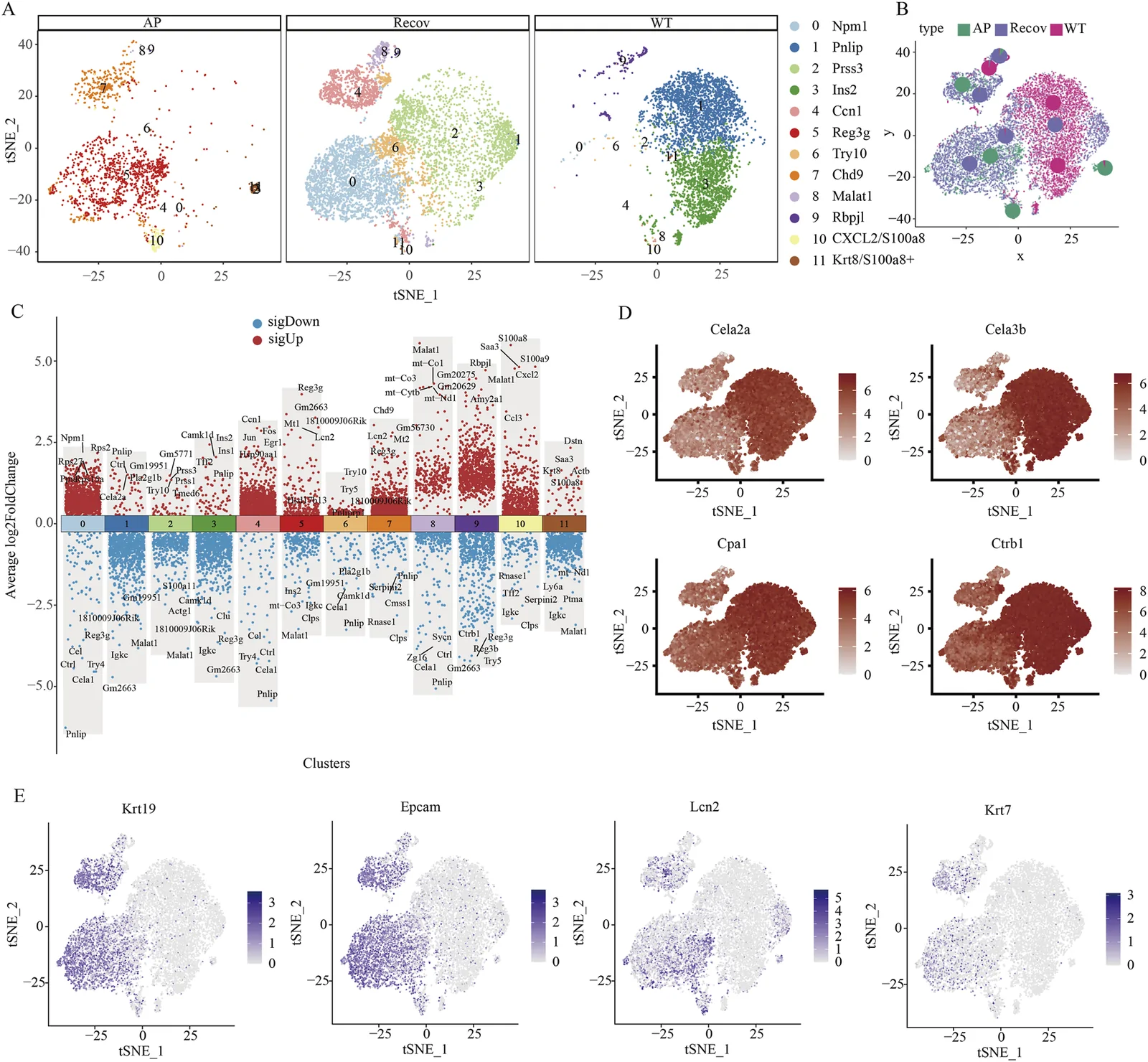
Secondary clustering analysis reveals heterogeneity and ductal-like features of acinar cells during acute pancreatitis and recovery. **(A)** t-SNE visualization of acinar cell subclusters after extraction and re-clustering from the integrated single-cell dataset. **(B)** t-SNE plot showing the distribution of acinar cell subclusters across different experimental groups, with pie charts indicating the relative contribution of AP, Recovery, and WT cells to each subcluster. **(C)** Differentially expressed marker genes across acinar cell subclusters. **(D)** Feature plots showing the expression of canonical acinar cell markers, including Cela2a, Cela3b, Cpa1, and Ctrb1, in the acinar cell t-SNE space. **(E)** Feature plots showing the expression of ductal- and injury-associated genes, including Krt19, Epcam, Lcn2, and Krt7, in acinar cell subclusters.

To validate the acinar identity of these subclusters, we examined the expression of canonical acinar markers. Cela2a, Cela3b, Cpa1, and Ctrb1 were broadly expressed across acinar subclusters, particularly in clusters enriched in WT and Recovery samples, confirming the acinar origin of the analyzed cells (Figure 2D). Notably, ductal-associated genes, including Krt19, Epcam, Lcn2, and Krt7, were selectively elevated in specific acinar subclusters, especially those enriched in AP and Recovery samples (Figure 2E). This finding indicates that a subset of acinar cells acquired ductal-like transcriptional features during acute pancreatitis and recovery, suggesting the presence of acinar cell plasticity and potential acinar-to-ductal metaplasia-like transition.

### Trajectory analysis reveals dynamic state transitions of acinar cells during pancreatic injury and recovery

To further explore the potential state transitions among acinar cell subclusters, pseudotime trajectory analysis was performed using Monocle2. The analysis reconstructed a branched trajectory structure, indicating that acinar cells followed divergent transcriptional programs during disease progression and recovery (Figure 3A). When cells were colored by experimental group, WT, AP, and Recovery cells showed distinct but partially overlapping distributions along the trajectory, suggesting dynamic remodeling of acinar cell states across different disease stages (Figure 3B).

**FIGURE 3:**
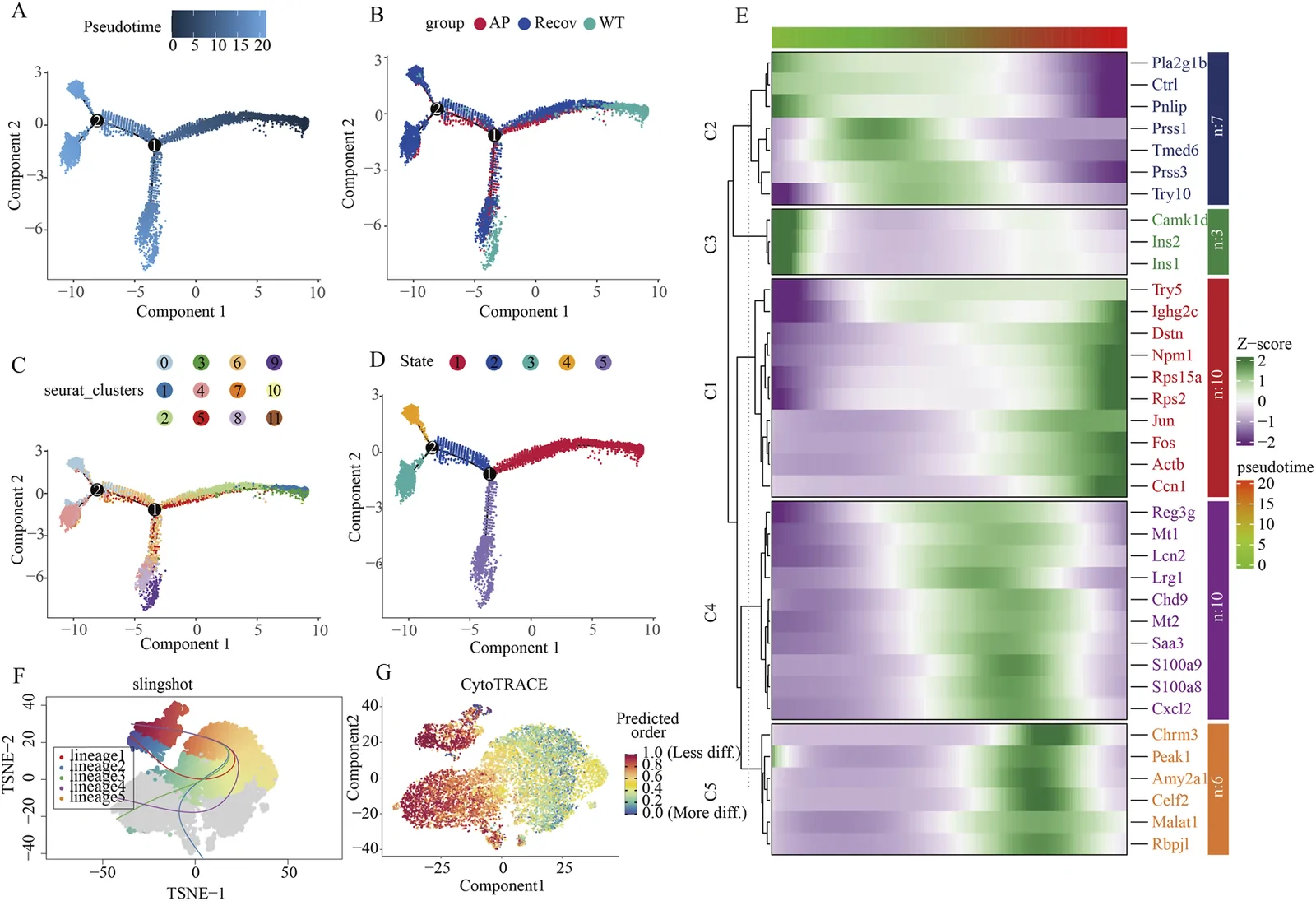
Trajectory analysis reveals dynamic state transitions of acinar cells during acute pancreatitis and recovery. **(A)** Monocle2 pseudotime trajectory of acinar cells colored by pseudotime. **(B)** Monocle2 trajectory of acinar cells colored by experimental group, including acute pancreatitis (AP), recovery, and wild-type (WT) groups. **(C)** Distribution of acinar cell subclusters along the Monocle2-inferred trajectory. **(D)** Monocle2 trajectory showing different cell states identified during acinar cell state transition. **(E)** Heatmap showing the dynamic expression patterns of representative acinar cell marker genes along pseudotime. **(F)** Slingshot trajectory inference showing multiple potential lineage paths among acinar cell subclusters. **(G)** CytoTRACE analysis showing the predicted differentiation order of acinar cells, with higher scores indicating less differentiated states and lower scores indicating more differentiated states.

Mapping acinar subclusters onto the pseudotime trajectory revealed that different subclusters occupied distinct positions along the inferred developmental or state-transition axis (Figure 3C). In addition, Monocle2 divided the trajectory into multiple states, further supporting the presence of heterogeneous acinar cell states during acute pancreatitis and recovery (Figure 3D). These results suggest that acinar cells do not undergo a uniform transcriptional response after injury, but instead transition through multiple intermediate and terminal states.

To characterize gene expression dynamics along pseudotime, representative marker genes were analyzed and grouped according to their temporal expression patterns (Figure 3E). Genes associated with mature digestive enzyme-producing acinar cells, such as Pla2g1b, Ctrl, Pnlip, Prss1, Prss3, and Try10, were enriched in specific pseudotime regions, whereas genes related to stress response, inflammation, and tissue remodeling, including Jun, Fos, Reg3g, Lcn2, Saa3, S100a8, S100a9, and Cxcl2, exhibited distinct dynamic expression patterns along the trajectory. These findings indicate that acinar cells progressively shift from a homeostatic digestive enzyme-secreting state toward injury-associated and inflammatory transcriptional states during acute pancreatitis.

To validate the robustness of the inferred acinar cell trajectory, Slingshot was further applied for lineage inference. Slingshot identified multiple lineage curves across the acinar cell landscape, supporting the presence of branched state-transition paths among acinar subclusters (Figure 3F). In addition, CytoTRACE analysis was used to estimate the differentiation potential of acinar cells. Cells with higher predicted developmental potential were mainly distributed in specific injury-associated regions, whereas more differentiated cells were enriched in regions corresponding to mature acinar populations (Figure 3G). Together, these trajectory analyses suggest that acute pancreatitis induces pronounced acinar cell plasticity, characterized by loss of mature acinar identity, activation of inflammatory and stress-related programs, and emergence of ductal-like transitional states during injury and recovery.

### Functional enrichment analysis reveals distinct metabolic and inflammatory programs among acinar cell subclusters

Functional enrichment analysis further revealed broad functional heterogeneity among acinar cell subclusters. KEGG analysis showed that different subclusters were enriched in metabolic pathways, including amino acid metabolism, lipid metabolism, carbohydrate metabolism, pyruvate metabolism, and oxidative phosphorylation, as well as immune-related pathways such as JAK-STAT, Toll-like receptor, RIG-I-like receptor, and NOD-like receptor signaling (Figure 4A). Hallmark analysis further identified cluster-specific activation of oxidative phosphorylation, glycolysis, fatty acid metabolism, inflammatory response, IL6-JAK-STAT3 signaling, TNF-α/NF-κB signaling, complement, interferon responses, reactive oxygen species pathway, apoptosis, and hypoxia (Figure 4B). GO enrichment analysis supported these findings, showing that acinar subclusters were associated with biological processes related to protein processing, endoplasmic reticulum stress, zymogen activation, digestion, antimicrobial response, inflammatory regulation, RNA processing, ribosome biogenesis, and cellular respiration (Supplementary Figure S1). Cellular component and molecular function analyses further highlighted enrichment of secretory granules, zymogen granules, endoplasmic reticulum, lysosomal and proteasomal compartments, ribosomes, mitochondria, serine-type peptidase activity, lipase activity, ubiquitin-related binding, and RNA-binding functions (Supplementary Figures S2, S3). These results indicate that acinar cells undergo marked functional diversification during acute pancreatitis and recovery, involving metabolic remodeling, stress responses, inflammatory activation, and altered digestive enzyme-associated programs.

**FIGURE 4:**
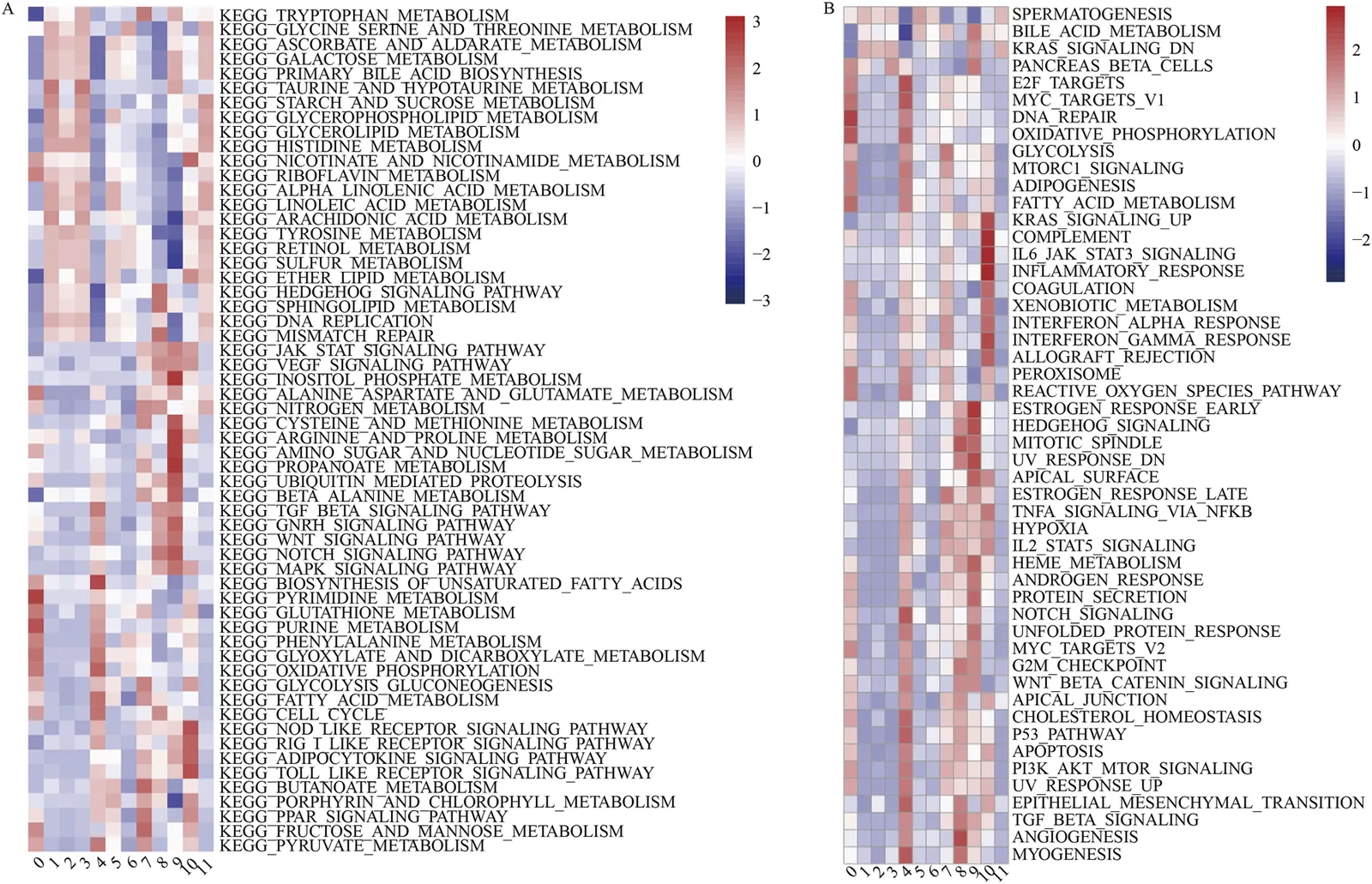
Pathway activity analysis reveals functional heterogeneity among acinar cell subclusters. **(A)** Heatmap showing KEGG pathway activity across 12 acinar cell subclusters. Pathways related to amino acid metabolism, lipid metabolism, carbohydrate metabolism, oxidative phosphorylation, inflammatory signaling, and innate immune signaling showed distinct enrichment patterns among different subclusters. **(B)** Heatmap showing Hallmark pathway activity across acinar cell subclusters. Inflammatory and stress-related pathways, including inflammatory response, IL6-JAK-STAT3 signaling, TNF-α signaling via NF-κB, interferon responses, reactive oxygen species pathway, apoptosis, and hypoxia, displayed subcluster-specific activation. Color intensity represents the scaled pathway activity score.

### Cell death-related scoring reveals PANoptosis-associated programs in acinar cell subclusters

To determine which specific acinar subclusters were associated with distinct forms of cell death, we separately evaluated pyroptosis, apoptosis, and necroptosis signatures using six scoring strategies. Pyroptosis activity was most prominent in Cluster 10, characterized by CXCL2/S100a8 expression, and was also increased in Cluster 7 and Cluster 4, suggesting that inflammatory acinar subclusters were preferentially associated with pyroptotic programs (Supplementary Figure S4A). Apoptosis scores showed a similar but broader distribution. Cluster 10 exhibited the highest apoptotic activity, whereas Clusters 7, 5, and 4 also showed elevated scores across multiple algorithms (Supplementary Figure S4B). In contrast, mature digestive enzyme-enriched clusters, such as Clusters 1, 2, and 6, displayed relatively lower apoptotic activity. Necroptosis-related signatures were mainly enriched in Clusters 10 and 7, with moderate activation in Clusters 4 and 5 (Supplementary Figure S4C). These subclusters largely overlapped with the pyroptosis- and apoptosis-enriched populations, indicating that inflammatory and injury-associated acinar cells were not restricted to a single type of programmed cell death. Together, these results suggest that Cluster 10, followed by Clusters 7, 4, and 5, represents the major acinar cell populations associated with enhanced cell death activity during acute pancreatitis and recovery.

We then focused on PANoptosis, an integrated inflammatory cell death program involving crosstalk among pyroptosis, apoptosis, and necroptosis. PANoptosis scores calculated by the six algorithms showed clear variation across the 12 acinar subclusters (Figure 5A). Notably, several injury-associated acinar subclusters displayed higher PANoptosis activity, whereas subclusters enriched in WT samples showed relatively lower scores. Consistent with the dot plot analysis, t-SNE visualization demonstrated that PANoptosis-related activity was spatially concentrated in specific acinar subcluster regions (Figures 5B–G). This pattern was observed across individual scoring methods, including AddModuleScore, AUCell, singscore, UCell, and ssGSEA, and was further supported by the integrated scoring strategy.

**FIGURE 5:**
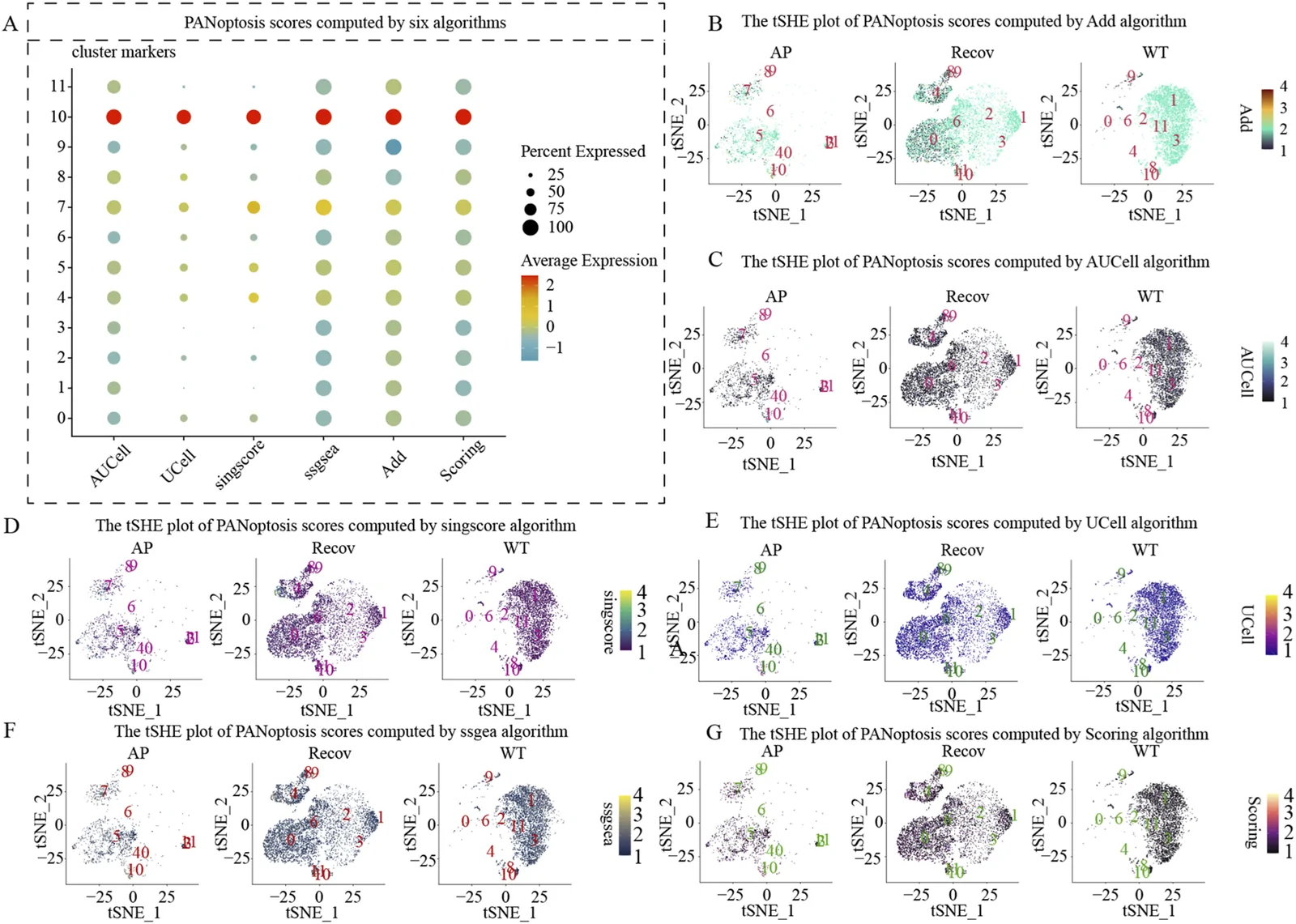
PANoptosis-related scoring analysis in acinar cell subclusters. **(A)** Dot plot showing PANoptosis scores across acinar cell subclusters calculated using multiple scoring strategies, including AUCell, UCell, singscore, ssGSEA, AddModuleScore, and the integrated Scoring method. Dot size represents the percentage of cells expressing the corresponding score, and color indicates the average score level. **(B)** t-SNE feature plots showing PANoptosis scores calculated by the AddModuleScore method in the acute pancreatitis (AP), recovery, and wild-type (WT) groups. **(C)** t-SNE feature plots showing PANoptosis scores calculated by the AUCell method across the AP, Recovery, and WT groups. **(D)** t-SNE feature plots showing PANoptosis scores calculated by the singscore method across the AP, Recovery, and WT groups. **(E)** t-SNE feature plots showing PANoptosis scores calculated by the UCell method across the AP, Recovery, and WT groups. **(F)** t-SNE feature plots showing PANoptosis scores calculated by the ssGSEA method across the AP, Recovery, and WT groups. **(G)** t-SNE feature plots showing the integrated PANoptosis score across the AP, Recovery, and WT groups. Higher scores indicate stronger PANoptosis-related activity.

When stratified by experimental group, PANoptosis-associated signals were more evident in AP and Recovery samples than in WT samples, suggesting that acute pancreatic injury induces persistent activation of inflammatory cell death programs in acinar cells. Together, these results indicate that acinar cell death during acute pancreatitis is not limited to a single mode of programmed cell death. Instead, distinct acinar subclusters exhibit coordinated activation of apoptosis-, necroptosis-, and pyroptosis-related signatures, supporting the involvement of PANoptosis-associated transcriptional programs in acinar cell injury and remodeling.

### Macrophage subclustering identifies PANoptosis-active inflammatory macrophage subsets

To investigate macrophage heterogeneity during acute pancreatitis and recovery, macrophages were extracted from the integrated single-cell dataset and subjected to secondary clustering analysis. Six macrophage subclusters were identified and displayed distinct distributions among the AP, Recovery, and WT groups (Figure 6A). Cluster 0 was predominantly enriched in AP samples, whereas Cluster 1 was mainly observed in the Recovery group. In contrast, WT samples contained relatively fewer macrophages and were mainly distributed in limited subcluster regions, suggesting marked macrophage expansion and remodeling during acute pancreatitis and recovery.

**FIGURE 6:**
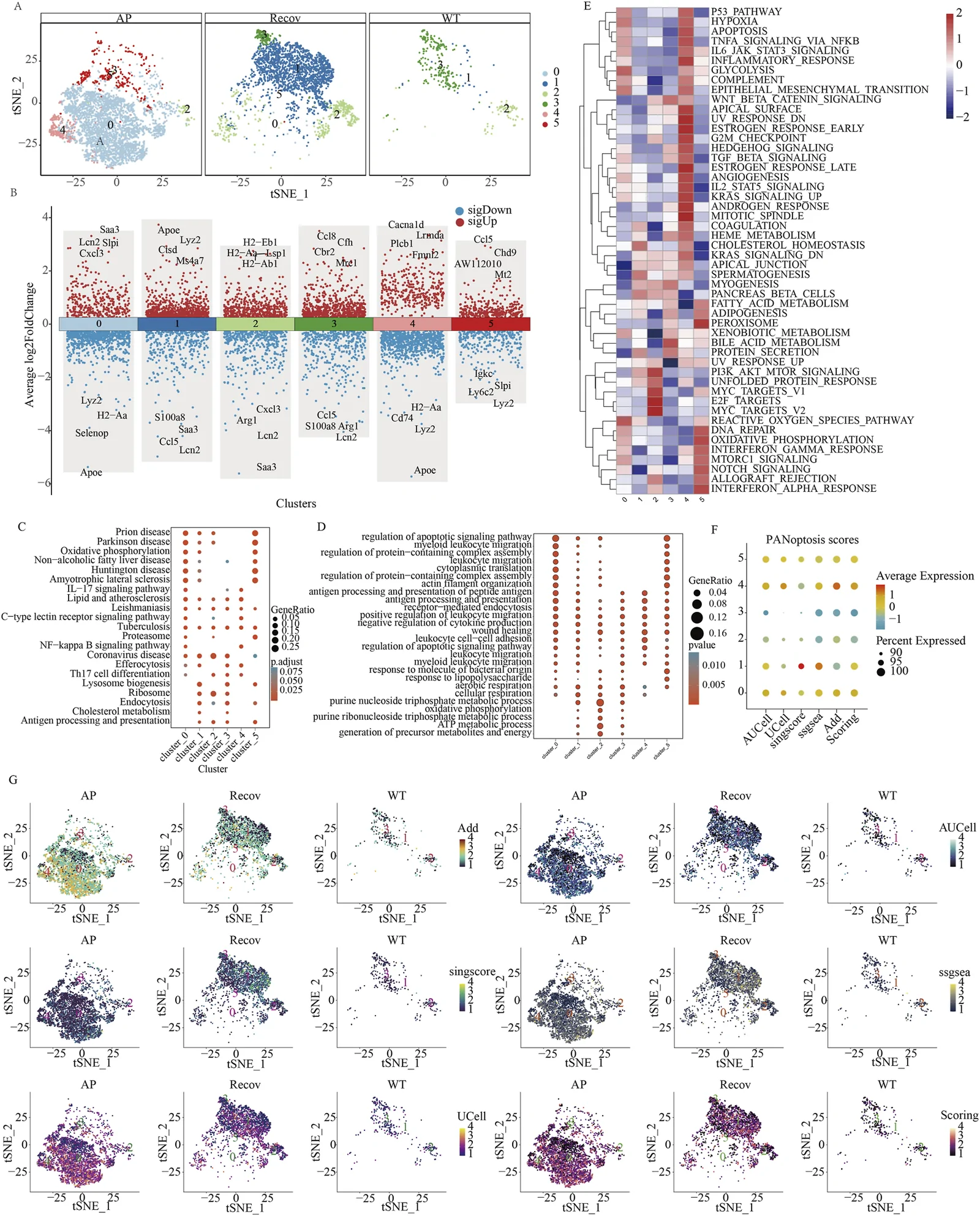
Macrophage subcluster analysis and PANoptosis-related scoring during acute pancreatitis and recovery. **(A)** t-SNE visualization of macrophage subclusters after extraction and re-clustering from the integrated single-cell dataset. **(B)** Differentially expressed marker genes across macrophage subclusters. **(C)** KEGG enrichment analysis of marker genes from macrophage subclusters. **(D)** GO biological process enrichment analysis of marker genes from macrophage subclusters. **(E)** Heatmap showing Hallmark pathway activity across macrophage subclusters. **(F)** Dot plot showing PANoptosis scores across macrophage subclusters calculated using AUCell, UCell, singscore, ssGSEA, AddModuleScore, and the integrated Scoring method. **(G)** t-SNE feature plots showing PANoptosis scores in macrophages calculated using AddModuleScore, AUCell, singscore, ssGSEA, UCell, and the integrated Scoring method across the AP, Recovery, and WT groups.

Differential expression analysis revealed distinct transcriptional signatures among macrophage subclusters (Figure 6B). Cluster 0 highly expressed inflammatory genes such as Saa3, Lcn2, and Cxcl3, indicating an inflammatory macrophage phenotype. Cluster 1 was characterized by Apoe, Lyz2, Ctsd, and Ms4a7, suggesting a phagocytic or reparative macrophage state. Cluster 2 showed increased expression of antigen-presentation-related genes, including H2-Eb1, H2-Aa, and H2-Ab1, whereas Cluster 3 expressed Ccl8 and Cfh, indicating chemokine- and complement-associated features. Cluster 4 was marked by genes such as Cacna1d, Lrmda, and Plcb1, while Cluster 5 showed increased expression of Ccl5, Chd9, and Mt2.

Functional enrichment analysis further supported the distinct biological properties of these macrophage subclusters. KEGG enrichment showed that macrophage subclusters were associated with immune and inflammatory pathways, including IL-17 signaling, lipid and atherosclerosis, C-type lectin receptor signaling, NF-κB signaling, lysosome, endocytosis, cholesterol metabolism, and antigen processing and presentation (Figure 6C). GO biological process analysis revealed enrichment of pathways related to regulation of apoptotic signaling, myeloid leukocyte migration, cytokine production, phagocytosis, antigen processing and presentation, leukocyte cell-cell adhesion, and ATP metabolic processes (Figure 6D). Hallmark pathway analysis also showed subcluster-specific activation of inflammatory and stress-related programs, including TNF-α signaling via NF-κB, IL6-JAK-STAT3 signaling, inflammatory response, hypoxia, apoptosis, complement, glycolysis, and epithelial-mesenchymal transition-related signatures (Figure 6E).

We next evaluated PANoptosis-related activity across macrophage subclusters using six scoring approaches. The dot plot showed that Cluster 4 exhibited the strongest PANoptosis activity, with consistently high scores across multiple algorithms, including singscore, ssGSEA, AddModuleScore, and the integrated scoring method (Figure 6F). Clusters 0 and 1 also showed relatively elevated PANoptosis scores, suggesting that both AP-enriched inflammatory macrophages and Recovery-enriched phagocytic/reparative macrophages were associated with increased inflammatory cell death signaling. Cluster 5 displayed moderate PANoptosis activity, whereas Clusters 2 and 3 showed comparatively lower PANoptosis scores, indicating that antigen-presenting and chemokine/complement-associated macrophage subsets were less strongly associated with PANoptosis. Consistently, t-SNE visualization of PANoptosis scores showed that high-score macrophages were mainly distributed in AP and Recovery samples, while WT macrophages exhibited relatively low PANoptosis activity (Figure 6G).

### Neutrophil subclustering identifies a PANoptosis-active inflammatory neutrophil subset

To further characterize neutrophil heterogeneity during acute pancreatitis and recovery, neutrophils were extracted from the integrated single-cell dataset and subjected to secondary clustering analysis. Five neutrophil subclusters were identified, and their distribution differed markedly among the AP, Recovery, and WT groups (Figure 7A). Neutrophils were predominantly enriched in the AP group, whereas only a small number of neutrophils were detected in WT samples, consistent with acute inflammatory infiltration during pancreatitis. The neutrophil identity of these subclusters was confirmed by the expression of canonical neutrophil markers, including S100a8, S100a9, Cxcr2, Fcgr3, and Itgam (Figure 7B). Differential expression analysis further revealed distinct transcriptional features among neutrophil subclusters (Figure 7C).

**FIGURE 7:**
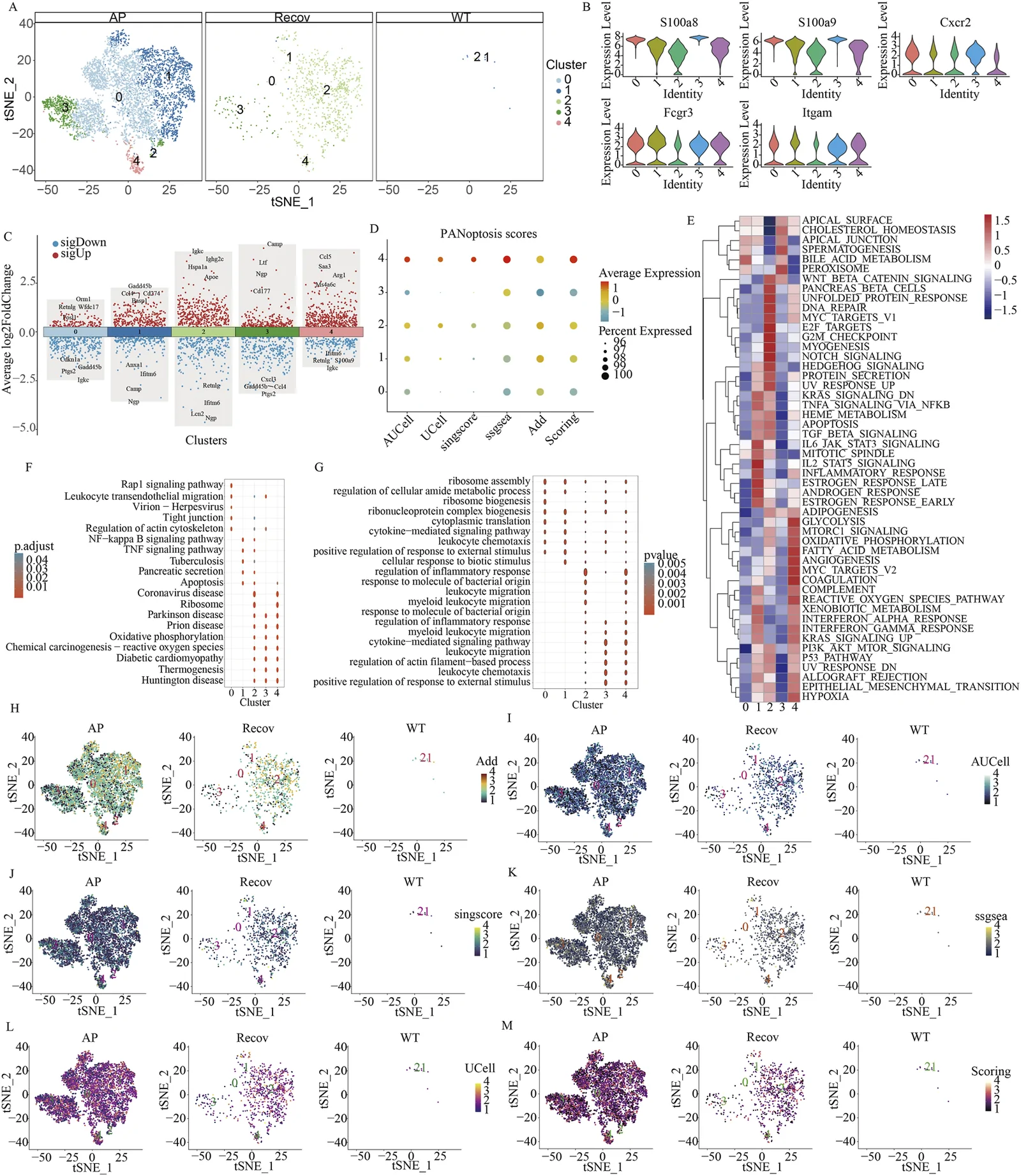
Neutrophil subcluster analysis and PANoptosis-related scoring during acute pancreatitis and recovery. **(A)** t-SNE visualization of neutrophil subclusters after extraction and re-clustering from the integrated single-cell dataset. **(B)** Violin plots showing the expression of canonical neutrophil markers, including S100a8, S100a9, Cxcr2, Fcgr3, and Itgam, across neutrophil subclusters. **(C)** Differentially expressed marker genes across neutrophil subclusters. **(D)** Dot plot showing PANoptosis scores across neutrophil subclusters calculated using AUCell, UCell, singscore, ssGSEA, AddModuleScore, and the integrated Scoring method. **(E)** Heatmap showing Hallmark pathway activity across neutrophil subclusters. **(F)** KEGG enrichment analysis of marker genes from neutrophil subclusters. **(G)** GO biological process enrichment analysis of marker genes from neutrophil subclusters. **(H–M)** t-SNE feature plots showing PANoptosis scores in neutrophils calculated using AddModuleScore, AUCell, singscore, ssGSEA, UCell, and the integrated Scoring method across the AP, Recovery, and WT groups.

Hallmark pathway analysis showed cluster-specific pathway activation among neutrophil subclusters (Figure 7E). Inflammatory and stress-related pathways, including inflammatory response, IL6-JAK-STAT3 signaling, TNF-α/NF-κB signaling, apoptosis, hypoxia, reactive oxygen species pathway, and interferon responses, were differentially enriched across neutrophil subsets. KEGG enrichment analysis further identified immune- and inflammation-related pathways, including leukocyte transendothelial migration, regulation of actin cytoskeleton, NF-κB signaling, TNF signaling, apoptosis, oxidative phosphorylation, and ribosome-related pathways (Figure 7F). GO biological process enrichment analysis also highlighted neutrophil functions related to leukocyte chemotaxis, cytokine-mediated signaling, response to bacterial molecules, regulation of inflammatory response, myeloid leukocyte migration, and ribosome biogenesis (Figure 7G).

We next assessed PANoptosis-related activity across neutrophil subclusters using multiple scoring algorithms. The dot plot showed that Cluster 4 exhibited the highest PANoptosis activity, with consistently elevated scores across ssGSEA, AddModuleScore, UCell, and the integrated scoring method (Figure 7D). Cluster 2 showed moderate PANoptosis activity, whereas Clusters 0, 1, and 3 displayed relatively lower scores. These results suggest that PANoptosis activation was not uniformly distributed across neutrophils but was preferentially enriched in a specific inflammatory neutrophil subset. Finally, PANoptosis scores were mapped onto the t-SNE space using different algorithms, including AddModuleScore, AUCell, singscore, ssGSEA, UCell, and the integrated scoring strategy (Figures 7H–M). Together, these findings indicate that Cluster 4 represents a PANoptosis-high inflammatory neutrophil subset, which may contribute to neutrophil-mediated inflammatory injury during acute pancreatitis.

## Discussion

Acinar cell demise represents a central pathological event driving the initiation and progression of acute pancreatitis; however, the molecular mechanisms underlying this process remain incompletely understood (Zhao J. et al., 2026). Increasing evidence (Zhang et al., 2026) indicates that AP is accompanied by the activation of diverse regulated cell death pathways, including apoptosis, necroptosis, pyroptosis, and ferroptosis, rather than being governed by a single dominant death modality. The coexistence and extensive crosstalk among these cell death programs suggest the presence of an integrated regulatory framework that coordinates inflammatory cell fate decisions. PANoptosis has recently emerged as a novel concept describing a coordinated form of inflammatory programmed cell death that integrates key features of apoptosis, necroptosis, and pyroptosis, providing a broader mechanistic perspective for understanding complex tissue injury in inflammatory diseases (Zhao M. et al., 2026; Wang et al., 2025). Based on single-cell transcriptomic profiling of mouse acute pancreatitis (WT, AP, and Recovery groups), this study reveals that acinar cells, macrophages, and neutrophils undergo extensive subpopulation remodeling during the injury and early repair phases, collectively converging on an inflammatory cell death network centered on PANoptosis. These findings not only expand the current framework of acute pancreatitis by revealing the dynamic interactions among acinar cell injury, immune responses, and tissue remodeling, but also suggest that targeting PANoptosis-high cellular subsets may provide a potential therapeutic avenue.

AP was characterized by a lower acinar-cell proportion and marked macrophage and neutrophil infiltration. These changes were only partially reversed in Recover, indicating persistent immune remodeling and tissue adaptation. Acinar cells occupied a continuum of mature secretory, injury-associated, inflammatory, and ductal-like transcriptional states. Macrophages and neutrophils also formed subsets with distinct inflammatory, phagocytic/reparative, and PANoptosis-associated programs. PANoptosis-related signatures were concentrated in specific subsets rather than uniformly distributed, supporting cell type- and state-specific regulation of inflammatory cell-death programs. Additionally, we observed a marked increase in the proportion of B cells during the recovery phase, suggesting that B cells may contribute to pancreatic repair. However, current evidence regarding the pro-reparative role of B cells in acute pancreatitis remains limited, and further investigations are warranted to elucidate the underlying mechanisms. Regarding ductal cells, we found that their proportion was significantly increased during AP-associated inflammatory injury, which may be closely related to extensive acinar-to-ductal metaplasia (ADM). The role of ductal cells in regulating pancreatic repair remains poorly understood, and further studies are needed to clarify how ductal cell responses contribute to tissue regeneration and recovery following pancreatic injury.

These findings are largely consistent with the current understanding of acute pancreatitis pathogenesis (Xia et al., 2024). It is widely accepted that the initiation of AP occurs primarily within pancreatic acinar cells, where intracellular calcium overload, mitochondrial dysfunction, excessive reactive oxygen species (ROS) accumulation, premature activation of digestive zymogens, and subsequent release of damage-associated molecular patterns collectively initiate local and systemic inflammatory responses (Chen X. et al., 2025; Pallagi et al., 2020). Increasing evidence (Zhang et al., 2026; Chen et al., 2024; Zhou et al., 2024) suggests that mitochondrial dysfunction represents a critical hub linking cellular stress responses and regulated cell death pathways. As a central organelle involved in cellular homeostasis, mitochondria function not only as sensors of intracellular stress but also as amplifiers of inflammatory signaling by regulating mitochondrial outer membrane permeabilization, mitochondrial DNA release, and ROS production. These processes can influence the balance among apoptosis, necroptosis, and pyroptosis, thereby shaping the inflammatory consequences of cell death. Crosstalk among these pathways, such as inflammasome-mediated mitochondrial damage and RIPK3-dependent metabolic reprogramming that enhances oxidative stress, highlights the existence of an interconnected regulatory network rather than isolated death modalities.

Our single-cell analysis further refined this classical framework by demonstrating that acinar cell responses to injury are highly heterogeneous. Rather than undergoing a uniform fate transition, only specific acinar subpopulations progressed toward injury-associated, inflammatory, and PANoptosis-related states, whereas others retained features of functional preservation or recovery. Similarly, immune cell infiltration during AP was not simply a quantitative increase in inflammatory cells, but involved the emergence of distinct macrophage and neutrophil subsets with divergent inflammatory and reparative characteristics. This cellular state heterogeneity may represent a critical determinant of disease progression and resolution, providing a more comprehensive view of how the balance between inflammatory amplification and tissue repair is established during acute pancreatitis.

Acinar cell subcluster analysis represents one of the key highlights of this study. We identified acinar cell populations exhibiting increased expression of ductal-associated genes, including Krt19, Epcam, Lcn2, and Krt7, during both the acute injury and early recovery phases of AP. Pseudotime analysis further revealed a potential transition trajectory from mature digestive enzyme-enriched acinar states toward stress-associated and ductal-like phenotypes. These findings are highly consistent with previous studies demonstrating acinar cell plasticity and acinar-to-ductal reprogramming during pancreatic injury (Yuan et al., 2026). Under pathological stress conditions, acinar cells can undergo dedifferentiation and lineage remodeling, which may represent an adaptive response facilitating tissue regeneration and repair, but may also contribute to pathological transformation under persistent inflammatory stimulation.

The prominence of the Prss3-marked Cluster 2 in Recovery may reflect re-establishment of an acinar digestive enzyme program during early repair. Prss3 encodes a trypsinogen-family serine protease; increased transcription could help restore digestive protease capacity as acinar secretory function recovers. However, greater Prss3 expression should not be assumed to be uniformly protective, because premature intracellular trypsinogen activation can aggravate acinar injury. Its effect is likely to depend on subcellular localization, zymogen activation, endogenous inhibitor balance, and secretion. Protein-level localization, enzyme-activity measurements, and targeted perturbation will therefore be required to determine whether Prss3 actively promotes repair or simply marks a recovering secretory state.

Importantly, we found that ductal-like reprogramming was not an isolated event but occurred together with the upregulation of injury- and inflammation-associated genes, including Reg3g, Jun, Fos, Lcn2, Saa3, and S100a8/S100a9. These observations suggest that acinar cell plasticity is closely interconnected with inflammatory stress responses and the activation of cell death-related programs. Therefore, acinar cell fate remodeling during AP may represent a complex adaptive process in which regenerative potential and inflammatory injury are simultaneously regulated.

Regarding cell death mechanisms, our findings support the interpretation of acute pancreatitis within the framework of PANoptosis. In acinar cells, we observed overlapping increases in pyroptosis-, apoptosis-, and necroptosis-related signatures, which ultimately converged into injury-associated subpopulations exhibiting high PANoptosis activity. Similar patterns were also identified in specific macrophage and neutrophil subsets. These findings are highly consistent with the concept of PANoptosis as an integrated inflammatory cell death program and are further supported by previous studies demonstrating that cytokine-driven inflammatory signaling can synergistically induce cell death and tissue injury. Therefore, we propose that cell death during acute pancreatitis is more likely characterized by a “networked mixed cell death program” rather than being dominated by a single specific cell death modality.

Nevertheless, several limitations of this study should be acknowledged. First, the sample size remains relatively limited, and the use of pooled samples from three mice for library construction, although increasing the total number of captured cells, may reduce the ability to evaluate inter-individual variability. Second, our analysis was restricted to three major time points, including WT, AP, and early recovery stages, which may not fully capture the continuous dynamic processes from initial injury to complete tissue restoration. Third, the identification of PANoptosis in this study was primarily based on transcriptomic signature scoring and pathway inference rather than direct evidence of PANoptosome assembly, activation of key proteins such as caspases, RIPK/MLKL, and GSDMD, or ultrastructural characterization. Therefore, our findings reflect a state of “PANoptosis propensity” rather than definitive execution of terminal PANoptotic cell death. Finally, functional validation *in vivo* and *in vitro* is still required to determine the causal roles of specific cellular subsets in the progression and resolution of acute pancreatitis.

## Conclusion

In conclusion, this study utilized single-cell transcriptomic profiling of the mouse pancreas to systematically characterize the cellular landscape during the early injury and repair phases of acute pancreatitis (AP). Our findings reveal dynamic alterations in acinar cell fate remodeling, inflammatory cell reprogramming, and PANoptosis-associated molecular signatures during disease progression. These results not only provide new insights into the complex interplay between regulated cell death and immune responses in AP but also establish a theoretical foundation for the development of more precise and stratified therapeutic interventions in the future.

## Data Availability

The original contributions presented in the study are included in the article/Supplementary Material, further inquiries can be directed to the corresponding author.

## Supplementary material

SUPPLEMENTARY FIGURE S1 GO biological process enrichment analysis of acinar cell subclusters.

SUPPLEMENTARY FIGURE S2 GO cellular component enrichment analysis of acinar cell subclusters.

SUPPLEMENTARY FIGURE S3 GO molecular function enrichment analysis of acinar cell subclusters.

SUPPLEMENTARY FIGURE S4 Cell death-related scoring analysis in acinar cell subclusters. Dot plot showing **(A)** pyroptosis, **(B)** apoptosis, **(C)** necroptosis scores across acinar cell subclusters calculated using AUCell, UCell, singscore, ssGSEA, AddModuleScore, and the integrated Scoring method.
